# Impact of estrogen on IgG glycosylation and serum protein glycosylation in a murine model of healthy postmenopause

**DOI:** 10.3389/fendo.2023.1243942

**Published:** 2023-09-11

**Authors:** Priti Gupta, Tibor Sághy, Jauquline Nordqvist, Jonas Nilsson, Hans Carlsten, Karin Horkeby, Petra Henning, Cecilia Engdahl

**Affiliations:** ^1^ Department of Rheumatology and Inflammation Research, Sahlgrenska Academy at University of Gothenburg, Gothenburg, Sweden; ^2^ Department of Internal Medicine and Clinical Nutrition, Sahlgrenska Osteoporosis Centre and Centre for Bone and Arthritis Research, Institute of Medicine, Sahlgrenska Academy at University of Gothenburg, Gothenburg, Sweden; ^3^ SciLifeLab, University of Gothenburg, Gothenburg, Sweden; ^4^ Proteomics Core Facility, Sahlgrenska Academy, University of Gothenburg, Gothenburg, Sweden

**Keywords:** IgG, estrogen, IgG-glycosylation, sialylation, sialic acid, animal, post-menopause

## Abstract

**Introduction:**

The glycosylation of immunoglobulin (Ig) G regulates IgG interaction capability with Fc gamma receptors found in all immune cells. In pathogenic conditions, estrogen can impact IgG levels and glycosylation. Following menopause, when estrogen levels decline affecting the immune system and potentially leading to a heightened susceptibility of immune activation.

**Purpose:**

In this study, we aim to determine if estrogen levels can regulate IgG glycosylation in postmenopausal healthy situations.

**Methods:**

Mice were ovariectomized to simulate an estrogen-deficient postmenopausal status and then treated with 17-beta-estradiol (E2) at different doses and different administration strategies.

**Results:**

Using a highly sensitive liquid chromatography-tandem mass spectrometry (MS/MS) glycoproteomic method, we demonstrated that E2 treatment increased the degree of glycosylation on IgG-Fc with both galactosylation and sialylation in the position required for interaction with Fc gamma receptors. We also observed that only long-term estrogen deficiency reduces IgG levels and that estrogen status had no impact on total IgG sialylation on both Fab and Fc domains or general glycoprotein sialylation evaluated by ELISA. Furthermore, E2 status did not affect the total sialic acid content of total cells in lymphoid organs and neither B cells nor plasma cells.

**Conclusion:**

The study concluded that E2 treatment does not affect total serum glycoprotein sialylation but alters IgG glycosylation, including IgG sialylation, implying that estrogen functions as an intrinsic modulator of IgG sialylation and could thereby be one pathway by which estrogen modulates immunity.

## 1 Introduction

Glycosylation is an important post-translational enzymatic modification that influences the folding, structure, and binding affinity of glycoproteins. N- and O-linked glycosylation are the two most common types of protein glycosylation. Glycans are well known for their ability to modify protein functions in several conditions, such as during infections, inflammation, aging, and tumor metastasis (1, 2).

Immunoglobulins (Ig) are the most common type of serum glycoproteins comprised mainly of IgG, IgM, and IgA. IgG is the most abundant antibody in serum and is essential for humoral immune responses. IgG is further classified into subclasses in mice IgG1, IgG2 (2a, 2b, and 2c), and IgG3, each with a varying binding affinity to fragment crystallizable gamma receptors (FcγRs). In mice, there are four different types of FcγRs: FcγRI, FcγRIIa, and FcγRIII are immune activating, whereas FcγRIIb is an inhibitory receptor (3, 4). The IgG is composed of four polypeptides, two identical light, and heavy chains. IgG structure is divided into two parts, the fragment antigen binding (Fab) region, and the fragment crystallizable (Fc) region, both parts are glycosylated. On the Fab part, both N- and O-linked glycosylation are present and involved in antigen-binding interaction. The Fc region only possesses a single conserved N-linked glycosylation site at Asn-297 in both heavy chains, contributing to the stability and the maintenance of the quaternary structure of IgG. The glycosylation of the IgG- Fc region affects the interaction capability with FcγRs, which has significant implications on antibody-dependent cell-mediated cytotoxicity, antibody-dependent cellular phagocytosis, the release of inflammation-associated mediator molecules and complement-dependent cytotoxicity (3, 4). Specifically, the terminal galactose and sialic acid (in the form of *N*-glycolylneuraminic acid (Neu5Gc) in mice), have been associated with anti-inflammatory properties via their ability to inhibit the interaction with FcγRs (5). This regulation is associated with various diseases, such as autoimmune diseases, both in the induction phase as well as in the progression of the disease, representing both predisposition and a functional mechanism in the disease pathology (6). Furthermore, the anti-inflammatory activity of highly glycosylated IgG has been previously reported in several experimental studies (7–9). IgG Fc-glycosylation may also play an important role in the regulatory effects that appear after menopause when the sex hormone, estrogen, begins to decline as evidenced by several studies in both healthy individuals (10–14) and in pathogenic conditions (9, 15–18). Estrogen levels are correlated with an increased IgG-Fc galactosylation and sialylation during pregnancy and rapid decrease after delivery, which has been directly associated with pregnancy-induced remission and a flare in disease activity following childbirth in rheumatoid arthritis patients (19–22).

Although several human epidemiological association studies have been conducted, further functional studies utilizing animal models are required to explore the regulatory mechanisms mediated by estrogen. Our study aims to investigate the impact of estrogen on IgG glycosylation and total glycoprotein sialylation in a normal postmenopausal murine model with either estrogen deprivation or estrogen treatment. This is to further understand whether estrogen treatment may boost immune function in normal postmenopausal women as well as the role of estrogen in disease development. Our study demonstrates that estrogenic treatment to ovariectomized mice had a significant impact on the IgG sialylationand a partial effect on galactosylation. This implies that estrogen modulates the sialylation of IgG also in helathy postmenopausal conditions, and this mechanism could be one of the pathways by which the hormone regulates the immune system.

## 2 Materials and methods

### 2.1 Animals and care

C57BL6 (Taconic) female mice were housed in a standard animal facility condition, Laboratory of Experimental Biomedicine at Gothenburg University, and fed phytoestrogen-free chow ad libitum (Harlan Rodent Diet, 2016).

### 2.2 Ovariectomy and hormone treatment

To avoid confounding endogenous sex hormone effect and mimic a postmenopausal state, mice were ovariectomized. Through a midline incision of the peritoneum followed by flank incisions, the ovary was removed, and the skin was closed with metallic clips. The same procedure was performed for sham surgery, except removal of the ovaries. The mice were sedated with isoflurane (Baxter Medical AB) and Metacam (Boehringer Ingelheim Animal Health) was used as preoperative analgesia. The sample size was calculated using the G*power software using data from a previous study for the sialic acid effect on IgG in the OVX-vehicle and OVX-E2 groups in the immune-induced mice (15). With an alpha value of 0.05, a power of 80%, and a calculated Cohen’s d effect size of 1.66, our study would require a sample size of 7 per group to attain significance.

**Experiment-I**- pellet lower dose of estradiol. Mice were subjected to OVX or sham surgery at nine weeks of age and terminated 36 days post-surgery. The slow-release subcutaneous (*s.c)* pellet was implanted during the OVX operation, containing 17-beta-Estradiol (E2) (167ng/day) or a placebo (Pla) (Innovative Research). In this experiment, 7-8 mice/group were used.
**Experiment-II**- injection higher dose of estradiol. Mice were subjected to OVX or sham surgery at nine weeks of age and terminated 36 days post-surgery. Treatment was initiated seven days post-surgery with *s.c.* injection of Pla (Miglyol 812; Omya Peralta GmbH) or 17beta-estradiol-3-benzoate (estradiol (E2) 1 µg/mice/day; Sigma Aldrich) five days per week of a total of four weeks. In this experiment, 10 mice/group were used.
**Experiment-III**- longer duration of estrogen deprivation. Mice were subjected to OVX or sham surgery at 12 weeks of age and left in estrogen deprivation status for a longer duration of 3 months and terminated on day 105 post-surgery. In this experiment, we used 10 mice/group.

On termination, all mice were sedated with Ketamine (Richter Pharma) and Dexmedetomidine (Orion Pharma Animal Health), blood was collected, and the mice were euthanized by cervical dislocation. Uterus, thymus, and gonadal fat were dissected and weighed.

### 2.3 Assessment of the bone

Mice tibia bone was dissected and fixed in 4% buffered paraformaldehyde for 48 hours followed by storage in 70% ethanol until subsequent bone mineral density (BMD) assessment. Trabecular BMD was assessed at the metaphyseal region of the tibia bone by pQCT scan analysis with Stratec pQCT XCT Research M software (software version 5.4; Norland, Fort Aktison, WI, USA) at a resolution of 70 µm as previously described (23). Cortical thickness was determined with a scan in the mid-diaphyseal region of the tibia bone with a scan of 36% from the growth plate.

### 2.4 Serum analyses

Blood samples were taken before surgery, during the experiment, and on the termination. Serum was obtained by centrifugation in the serum-gel tube (Sarsted, Sweden). Total serum IgM, IgGs (Bethyl Laboratories), and IgG-subtypes IgG1, IgG2a, and IgG2b were measured using commercially available ELISA kits (Thermo Fisher, Invitrogen) and read the plate using a microplate reader at 450 nm absorbance. The total sialic acids on the IgG were measured with Lectin ELISA, as described previously (15). Briefly, anti-mouse IgG F(ab)2 fragment (Sigma Aldrich, Sweden) was used to coat 96 well plates overnight at 4°C in sodium bicarbonate buffer (0.1M, pH 9.6), then blocked for 1-2 hours using polyvinyl pyrrolidone (0.5%) in warm Tris buffer saline buffer (TBS) (Sigma Aldrich-PVP10). Incubated with serum (1:100 dilution) in TBST (0.1% tween 20) followed by biotinylated Sambucus nigra lectin (SNA) (1:4000 dilution) (Vector Laboratories, Sweden) and then streptavidin-HRP (R&D Systems, Sweden) (1:1000 dilution) were used to detect sialylation followed by substrate addition and read the plate using a microplate reader at 450-620 nm absorbance.

### 2.5 IgG purification from murine serum

In a subset of ovariectomized mice from the experiment-II where an adequate volume of serum (240 µl) was available. Mouse IgG was purified using a commercially available IgG purification kit (Protein G High-Performance Spintrap™Sigma Aldrich) according to manufacturer protocol. Following purification, the concentration of IgG and protein were determined with a commercially available IgG ELISA kit (Bethyl Laboratories) and DC protein kit (Bio-rad). Purified IgG sample (30 μg protein) were reduced, Cys residues derivatized with *S*-Methyl methanethiosulfonate and cleaved in-solution with trypsin (1:50) in the presence of 0.5% sodium deoxycholate using standard procedures as described in (24). The samples were desalted using Pierce C18 desalting columns (Thermo Scientific) according to the manufacturer’s instructions.

### 2.6 Glycoproteomic analysis of IgG

An Exploris 480 Orbitrap instrument (Thermo Scientific) interfaced with an Easy-nLC-1200 chromatography system was used for the nano-LC-MS/MS analysis. The trapping column was an Acclaim Pepmap 100 C18 (Thermo Scientific) and the analytical column was in-house packed with Reprosil-Pur (75 μm × 350 mm, particle size 3 μm, Dr Maisch). The flow was set to 300 nL/min and the 90 min elution gradient was 5% B in A to 45% B in A over 78 min, then to 100% B over 2 min, and then kept at 100% B for 10 min. A was 0.2% formic acid in water, B was 80% acetonitrile, and 0.2% formic acid in water. Each sample (2 mL) was analyzed three times consecutively with different MS/MS settings, see below.

The precursor Ion MS1 spectra were collected at m/z 600-2200 at a mass resolution of 120 000. The most intense ions were subjected to MS/MS (MS2), using an isolation window of 3 m/z units, with higher-energy collision dissociation (HCD) at a normalized collision energy (NCE) of 20 for the first injection, NCE 30 for the second injection and NCE 38 for the third injection. The MS2 spectra were collected with a resolution of 30 000 with a first mass of m/z 110. The cycle time was 2 sec and an exclusion time of 12 sec was used.

The LC-MS/MS identification of trypsin-digested glycopeptides was done with the Byonic node (Protein Metrics) of Proteome Discoverer (v 2.4, Thermo Scientific) and the precursor ion intensities were extracted with the Minora Feature Detector node. Search settings included: the database was Swiss-Prot mouse (17 155 sequences); cleavage after Arg and Lys; two missed cleavages allowed; mass accuracy set to 10 ppm and 30 ppm at MS1 and MS2, respectively; fixed modification was a methylthio group at Cys; allowed modification was Met oxidation (set to rare 1); The N-glycan database (set to common 1) was selected to contain 123 complex type, oligomannose, and hybrid structures and included ones typically observed for immunoglobulins, and contained 0-3 of either sialic acid Neu5Ac or Neu5Gc glycoforms, 0-1 fucose, and 0-1 bisecting GlcNAc. The glycopeptide identities for each sample were filtered to include at least one identity from the three injections, which had a Byonic score >300. Glycopeptide hits were manually verified to contain the expected peptide+GlcNAc ion (Y1 ion) and if Fuc was suggested the peptide+GlcNAc+Fuc ion (core fucosylation), or an ion at m/z 512 (antenna fucosylation), had to be present. Further, identities containing NeuGc had to include the ions at m/z 290 and m/z 308; and correspondingly, m/z 274 and m/z 292 had to be present for Neu5Ac.

### 2.7 Relative quantitation of glycopeptides

The peak intensity ratio, in percentage, for each glycoform was calculated by dividing the Minora detected precursor ion intensity with the summed intensities of the identified glycoforms that share the same peptide and presented as average values for the three injections.

### 2.8 Quantification of total protein sialylation in serum

Total Sialic acid (Neu5Ac + Neu5Gc) was measured in total serum using the Sialic acid assay kit (Sigma-Aldrich, Sweden) according to manufacturer protocol. Total protein was measured using a DC protein assay kit (Bio-Rad, Sweden) according to manufacturer protocol. Protein sialylation was quantified by the ratio between total sialic acid and total protein.

### 2.9 Flow cytometry analyses

In experiment II, single-cell suspensions in PBS were prepared from bone marrow (BM) and spleen for flow cytometry analyses. BM cells were collected by flushing the BM in PBS followed by erythrolysis in 0.83 NH_4_Cl solution (pH 7.4). Splenocytes for flow cytometry were prepared by mashing the spleen tissue using a syringe and collecting the cells through 70 µM cell strainers in PBS, followed by erythrolysis in 0.83 NH_4_Cl solution (pH 7.4). Cells were counted using an automated cell counter (Sysmex Europe GmBH, Sweden). Flow cytometry analysis was performed using fluorochrome-conjugated antibodies i.e., APC anti-CD267 (TACI), PerCP-anti-CD19, BV421- anti-CD138, V500-anti-B220 and APCCy7-anti-CD3, all purchased from eBioscience (Sweden). Intracellular FITC-SNA (Vector lab, Sweden) was used to stain sialic acid. Analysis was performed using the BD FACS-verse flow cytometer and Flow Jo software (FlowJo10.6.2).

### 2.10 Isolation of total cellular RNA and quantitative PCR

Total RNA was extracted from BM, and spleen tissue with an RNeasy mini kit (Qiagen) and reverse transcribed into cDNA using a cDNA Reverse Transcription synthesis kit (Thermo Fisher Scientific). cDNA corresponding to 2.5-5 ng RNA was used for quantitative PCR with TaqMan (Thermo Fisher) and analysis for mRNA expression was performed with the Applied Biosystem Step OnePlus Real-Time PCR System using *ST6Gal1* (TaqMan Mm00486119_m1, NCBI Gene ID- 20440), *B4Galt2* (TaqMan Mm00480752_m1, NCBI Gene ID- 53418), and *Fut8* (TaqMan Mm00489795_m1, NCBI Gene ID- 53618) primers and probe set (Thermo Fisher) following standard TaqMan Assay protocol with a cycling condition of an initial cycle for reverse transcriptase at 50^°^C for 30 minutes, DNA polymerase activation at 95^°^C for 2 minutes, followed by 35-45 cycles of a 15-second denaturation at 95^°^C and then 60 seconds annealing and extension at 60^°^C. The housekeeping gene 18S (4310893E, Applied Biosystems) was used as an endogenous control in all analyses using the ddCT method and presented as % of the OVX-Pla group.

### 2.11 Statistical analysis

Statistical analyses were performed using GraphPad Prism software (version 0.1.0 (216)). Groups were compared with a one-way analysis of variance (ANOVA) for experiments- I and -II, followed by Dunnett’s multiple comparisons against the OVX-Pla group. Student´s *t*-test was used to compare the two groups. Grubb’s test was performed to detect significant outliers which were removed from further analysis. Data are presented as mean ± standard error of the mean (SEM) bar or as scatterplots, and p < 0.05 was considered statistically significant.

## 3 Results

### 3.1 Estrogen status has an impact on organ weight, bone marrow cellularity, and bone in mice

Successful ovariectomy was confirmed by a significant decrease in uteri weight compared to the sham group in all three experiments (**Table 1**). The mice that received E2 treatment had significantly larger uteri than those in the OVX-Pla group (**Table 1**). As expected, E2 treatment in OVX mice reduced thymus and gonadal fat weight as well as bone marrow cellularity compared to the OVX-Pla group (**Table 1**). Both short- and long-term estrogen deficiency affected gonadal fat weight and bone marrow cellularity compared to sham-operated mice. However, thymus weight was only significantly affected in the short term. Furthermore, we assessed the impact of E2 treatment effect on trabecular BMD and cortical thickness of the tibial bone. Our results showed that E2 treatment significantly increased both trabecular BMD and cortical thickness in OVX mice (**Supplementary Figures S1A, B**).

**Table 1:** Organ weight (mg) and bone marrow cellularity.

	Uterus	Thymus	Gonadal fat	Bone marrow cellularity (10^6^ cells)
Experiment-I				
Sham-Pla	83.8 ±18.76 **	43.0 ±6.05 **	325.1 ±46.70 **	25.87 ±3.15 ***
OVX-Pla	7.07 ±0.36	74.5 ±3.46	556.8 ±0.056	38.85 ±1.14
OVX-E2	118.7 ±13.74 ***	34.9 ±6.19 ***	261.9 ±17.30 ***	13.52 ±1.34 ***
Experiment-II				
Sham-Pla	71.3 ±10.02 ***	41.1 ±3.55 **	265.1 ±32.85 **	33.68 ±1.84 ***
OVX-Pla	9.0 ±0.46	58.2 ±3.18	382.52 ±24.12	44.85 ±1.85
OVX-E2	191.5 ±9.65 ***	20.5 ±4.40 ***	174.7 ±11.70 ***	22.16 ±2.13 ***
Experiment-III				
Sham	73.1 ±9.93	63.0 ±4.54	1443.5 ±169.22	20.27 ±1.33
OVX	10.16 ±0.87 ***	62.5 ±3.90	2495.2 ±224.77 **	24.43 ±0.98 *

Data expressed as mean ± SEM. Significant differences were calculated towards ovariectomized (OVX)-placebo (Pla) in experiments-I and -II using (One-way ANOVA) followed by Dunnet’s multiple comparison tests and in experiment-III student’s t-test was used. *P < 0.05, **P < 0.01, ***P < 0.001 represent significant differences vs the OVX-Pla group in experiments-I/II and vs the sham group in experiment-III.

### 3.2 Only long-term estrogen deficiency affects serum IgG levels and estrogen status does not affect total sialic acid present on IgG as detected by Lectin ELISA

**Figure 1:**
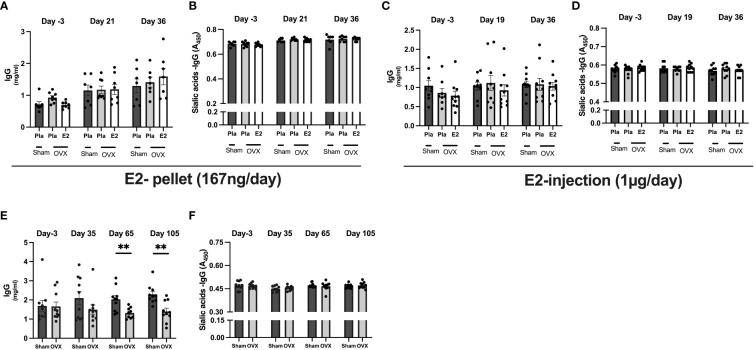
Estrogen does not influence immunoglobulins levels and sialic acid on IgG. Mice were ovariectomized (OVX), followed by the implantation of slow-release 17-β-estradiol (E2) and placebo (Pla) pellets (experiment-I), subcutaneous injection of E2 and Pla (experiment-II), and long-term estrogen-deficient (experiment-III). Serum was collected before OVX (day -3), on day 19/21 or day 35+ day 65 after OVX, and on day the end of experiments. Experiment-I; **(A)** total IgG concentrations, **(B)** sialic acid on IgG. Experiment-II; **(C)** total IgG concentrations, and **(D)** sialic acid on IgG. Experiment-III; **(E)** total IgG concentrations, and **(F)** sialic acid on IgG. Statistical analysis was performed with One-way analysis of variance (ANOVA) followed by Dunnett’s multiple comparison tests towards OVX-Pla group in experiments- I and II and student T-test for experiment-III. Data are presented as scattered bar graphs with mean ± SEM.**p < 0.001.

To assess the effect of estrogen on serum IgG levels and total sialic acid attached to IgG of both the Fab and Fc domain, we conducted serum analyses with ELISA in all experiments. The total IgG levels were not affected by OVX or E2 treatment in experiments- I and -II (**Figures 1A, C**). However, in long-term estrogen deficiency (after 65 days), OVX mice had significantly decreased serum IgG levels compared to sham-operated mice (**Figure 1E**). The levels of different IgG isotypes (IgG1, IgG2a, and IgG2b) in serum from termination in all experiments showed no significant variation dependent on estrogen status (**Supplementary Figures 2A–C**). However, long-term estrogen deficiency showed a strong tendency to lower the levels of IgG1, IgG2a, and IgG2b in a similar way as total IgG from the long-term healthy post-menopausal status. The same pattern was also displayed with IgM levels, with only long-term estrogen deprived in OVX mice displaying a strong tendency to reduce levels compared to sham mice (**Supplementary Figures S3A–C**). Lectin ELISA was used to assess the degree of total IgG sialylation on both the Fab and Fc domains, but no alteration was found in sialic acid on total IgG in any of the experiments (**Figures 1B, D, F**).

### 3.3 Estrogen treatment increased the sialylation of IgG in normal postmenopausal mice

**Figure 2:**
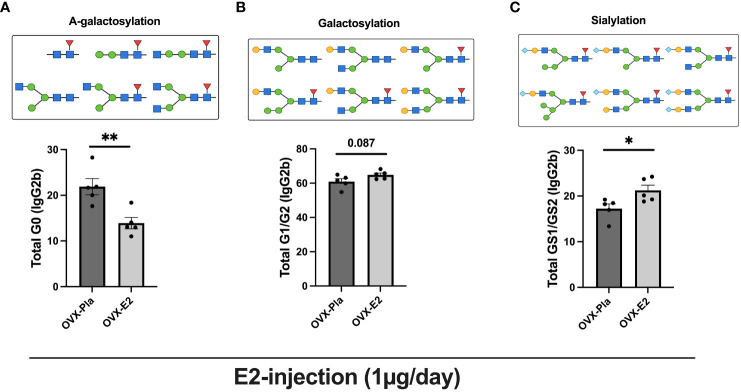
The LC-MS/MS method shows that estrogen increases the sialylation of IgG. Mice were ovariectomized (OVX), followed by the subcutaneous injection of 17-β-estradiol (E2) or placebo (Pla) (experiment-II). Mass spectrometry-based analysis of sialic acids and galactose on IgG subtypes, IgG2b (peptide sequence EDYNSTIR) were measured at termination 36 days after OVX (n = 5 mice/group, OVX-Pla and OVX-E2 group). **(A)** Percentage of G0 form as graphically displayed above on IgG2b (% of total IgG2b), **(B)** Percentage of total galactose G1/G2 form as graphically displayed above on IgG2b (%), and **(C)** Percentage of total sialic acid GS1/GS2 form as graphically displayed above on IgG2b (%). Values are indicated on a scattered bar graph with mean ± SEM. Student t-tests were used to calculate the statistics. G0: Agalactosylated, G1: mono-galactosylated, G2: di-galactosylated, G2S1: mono-sialylated, and G2S2: di-sialylated glycoforms. Symbols used: green circle: mannose, yellow circle: galactose, blue square: N-acetylglucosamine, red triangle: fucose, blue diamond: N-glycolylneuraminic acid (Neu5Gc). *p < 0.05 and **p < 0.001.

Next, we performed a comprehensive investigation of the higher dose of E2 (injection) treatment in the post-menopausal normal circumstances on IgG-Fc glycosylation using a highly sensitive LC-MS/MS-based glycoproteomic analysis, which detected 19 different glycoforms on the IgG-Fc region (**Supplementary Table S1**). Our analysis revealed that purified IgG from OVX mice from experiment II contained the highest levels of IgG2b subtype (Uniprot entry P01867, peptide sequence EDYNSTIR) and therefore we continued the investigation on this IgG subtype. OVX-E2-treated mice displayed a sharp decrease in A-galactosylated IgG, i.e., G0 glycoforms compared to the OVX-Pla group (**Figure 2A**). A strong tendency of further glycosylated IgG, carrying one and two galactose residues (G1/G2), was measured, in the OVX-E2 group (**Figure 2B**). Interestingly, the OVX-E2 group showed a significant increase in sialylated IgG-Fc GS1/GS2 glycoforms (**Figure 2C**). However, the core fucosylation of IgG remained unchanged between OVX-Pla and OVX-E2 groups (**Supplementary Table S1**).

### 3.4 Estrogen status does not influence intracellularly sialic acid in mice bone marrow

**Figure 3:**
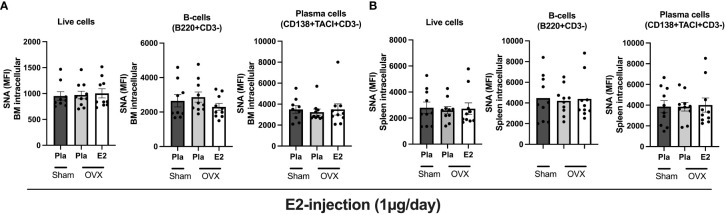
Estrogen impact on the intracellular sialic acid levels (SNA) in postmenopausal mice. Mice were ovariectomized (OVX), followed by the subcutaneous injection of 17-β-estradiol (E2) or placebo (Pla) (experiment-II). Bone marrow and spleen were collected for flow cytometry analyses. **(A)** Sialic acid levels in BM-derived total cells, B-cells, and plasma cells were measured by SNA staining. **(B)** Sialic acid levels in splenocyte-derived total cells, B-cells, and plasma cells were measured by SNA staining. MFI: mean fluorescence intensity. One-way ANOVA followed by Dunnett’s multiple comparisons to assess differences in the OVX-Pla group. Data are presented as scattered bar graphs with mean ± SEM.

Next, we wanted to see if estrogen treatment affects the sialic acid levels intracellularly in the bone marrow cells and splenocytes. We characterized the total live cells, B- cells, and plasma cells in the bone marrow cells and splenocytes from the experiment-II by performing flow cytometry analysis. We assessed the sialic acid levels by measuring the mean fluorescence intensity (MFI) after staining with sialic acid binding SNA. We found that neither estrogen removal (OVX) nor estrogen treatment affected the sialic acid levels in bone marrow or spleen-derived total live cells, B-cells, or plasma cells (**Figures 3A, B**).

### 3.5 Estrogen status does not impact serum protein sialylation in normal postmenopausal mice

**Figure 4:**
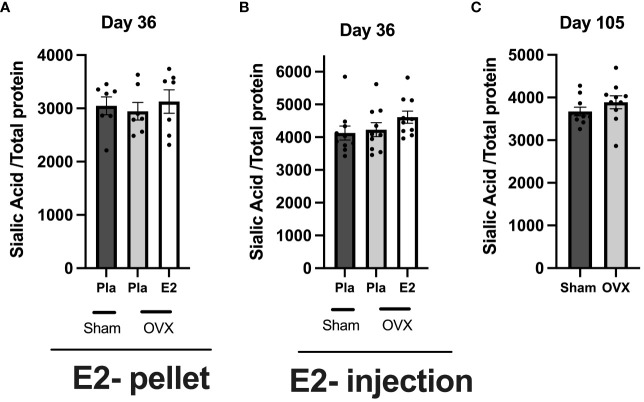
Estrogen does not influence total serum protein sialylation in normal postmenopausal mice. Mice were ovariectomized (OVX), followed by the insertion of slow-release 17-β-estradiol (E2) and placebo (Pla) pellets (experiment-I), subcutaneous injection of E2 and Pla (experiment- II), and long-term estrogen-deficient (experiment-III). Serum was collected at the end of the experiments. **(A)** Experiment-I sialic acid/total protein, **(B)** experiment-II sialic acid/total protein, and **(C)** experiment-III sialic acid/total protein. One-way ANOVA followed by Dunnett’s multiple comparisons to assess differences towards a OVX-Pla group in experiments-I and -II and student’s t-test were used in experiment-III. Data are presented as scattered bar graphs with mean ± SEM.

To determine the effect of estrogen on total serum glycoprotein sialylation, we quantified the sialic acid in total serum using ELISA in all three experiments. In healthy postmenopausal conditions, neither E2 treatment nor short- or long-term estrogen deficiency affects total glycoprotein sialylation (**Figures 4A–C**).

### 3.6 Estrogen treatment has an inhibitory effect on glycosyltransferase in bone marrow, and spleen tissue in ovariectomized mice

**Figure 5:**
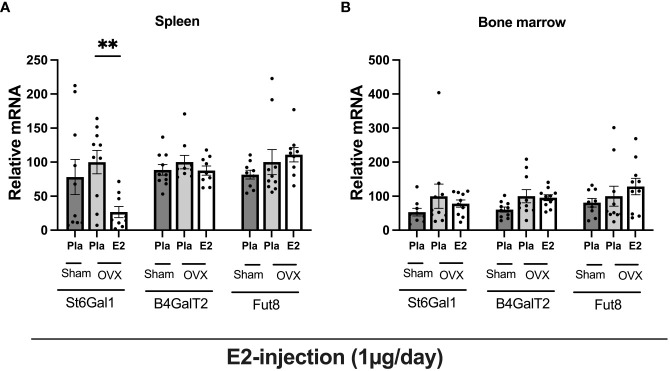
Estrogen treatment has an inhibitory effect on glycosyltransferase in bone marrow, and spleen tissue in ovariectomized mice. Mice were ovariectomized (OVX), followed by the subcutaneous injection of E2 and Pla (experiment- II). Bone marrow and spleen tissue were collected and purified total RNA, followed by glycosyltransferase mRNA measurement. **(A)** relative mRNA of spleen, and **(B)** relative mRNA of bone marrow. One-way ANOVA followed by Dunnett’s multiple comparisons to assess differences towards an OVX-Pla group. Data are presented as scattered bar graphs with mean ± SEM. **p < 0.001.

To determine the impact of estrogen status on the regulation of general glycosylation we analyzed the mRNA level of glycosyltransferases, sialyltransferase (*St6gal1)*, galactosyltransferase (*B4galt2)*, and fucosyltransferase (*Fut8)* in spleen tissue, and bone marrow (**Figure 5**) from the experiment- II. E2 treatment, significantly reduced *St6gal1* mRNA expression in the spleen, whereas *B4Galt2* and *Fut8* had no effect (**Figure 5A**). None of the glycosyltransferases were affected by E2 status in the bone marrow (**Figure 5B**).

## 4 Discussion

The relationship between sex steroids and the immune system is complex. Sex steroids have been found to modulate various immunological diseases as well as enhance the induction of systemic humoral immune induction (25–28). The effect of sex steroids on immune function is crucial to comprehend due to the alteration based on gender and life stage, such as puberty, pregnancy, and menopause in women (10). We have previously demonstrated that estrogen, but not the selective estrogen modulator Bazedoxifene, has an effect on IgG pathogenicity by altering the sialylation in immune-induced conditions (15, 18). Estrogen treatment to post-menopausal status upregulate sialyltransferase and increased levels of sialic acid in plasma cells (15). Extensive research has been dedicated to understanding antibodies and, more specifically, autoantibodies in autoimmune diseases like rheumatic arthritis. Our previous study has provided some evidence that the decrease in estrogen levels during menopause can potentially enhance the pathogenicity of antibodies. It is however important to recognize that antibodies play a vital role beyond autoimmune diseases as crucial supporting factors in immune system memory. In this context, the degree of glycosylation in antibodies assumes significant importance as it can impact their pathogenicity. Additionally, estrogen deficiency has been associated with alterations in the immune system, including an increase in serum pro-inflammatory cytokines, and a decrease in T and B lymphocytes. As a result, reduced estrogen levels contribute to a decrease in the immunological reactivity (26). Furthermore, the role of glycosylation in antibodies becomes even more important, potentially playing a crucial role in estrogen deficiency status.

Therefore, we hypothesized that estrogen might be one of the regulatory factors, influencing antibody pathogenicity also in healthy postmenopausal women. We found that estrogen treatment increased IgG-Fc sialylation (G1S/G2S form), decreased A-galactosylation (G0 form), and had a strong tendency towards increased galactosylation (G1/G2 form) on IgG, indicates in damping antibody pathogenicity in healthy postmenopausal women.

Estrogen affects the growth of estrogen-sensitive organs such as the uterus, gonadal fat, and thymus. In line with previous reports by (29–31), we showed that estrogen treatment increased the uterus weight and decreased the thymus and gonadal fat weight. Furthermore, estrogenic- effects were also observed in the bone, with reduced bone marrow cellularity, and increased trabecular BMD as well as cortical thickness which is consistent with previous studies (29, 30, 32, 33).

In previous studies, we have demonstrated that estrogen treatment increases IgG production in inflammatory conditions (15, 18). However, in the present study, we did not observe any difference in serum IgG levels depending on E2 status within the same time frame as our previous experiment. This suggests that estrogen treatment may not have an immediate effect on antibody production in a healthy non-inflammatory circumstance. Instead, estrogens impact on IgG production is more reliant on an activated immune system, such as during inflammation. Furthermore, long-term estrogen deficiency resulting from ovariectomy significantly reduced IgG levels even in healthy conditions, as well as showing a strong tendency to reduce levels of IgG1, IgG2a, IgG2b, and IgM. Not only estrogen is affected by ovariectomy, progesterone is dramatically reduced, however, progesterone levels have not previously displayed any alteration of Ig but this needs to be further investigated. These long-term findings contrast with *Surman et al.*, which demonstrated no significant change in serum Ig levels five months after ovariectomy (34). This suggests that the impact of estrogen loss may often be dependent on an activated immune system. The levels of IgG1 and IgG2a were low but detectable in the healthy OVX mice in contrast to the IgG2b levels which were higher, as measured by ELISA, and dominantly detected in the mass spectrometry analysis.

It is well known that IgG-Fc sialylation alters the immune activation of IgG. IgG-Fc sialylation has been shown to decrease the interaction capability with FcγRs which enhances the engulfment and pathogen destruction as well as complement activation thereby balancing a pro- and anti-inflammatory response (10). On the other hand, A-galactosylated IgG might activate the complement system via the Lectin pathway and increase the affinity to FcγRIII, an activating FcγR as well as interaction capability with various immune cells and may impact the immune response (10). Increased sialylation of IgG, on the other hand, decreases its affinity for FcγRIII while increasing the affinity with FcγRIIB, an inhibitory receptor. Large studies in human populations indicated that IgG glycans lacking galactose and sialic acid increase after menopause (35), but in younger females, they decrease with the onset of puberty (36, 37) as well as in pregnancy (38, 39), implying that estrogens may be involved in the alteration and thereby interaction capability with FcγRs. We and others have previously reported that E2 influences IgG-Fc sialylation in inflammatory conditions (15, 18, 40). In addition, estrogen treatment increases IgG-Fc glycosylation in normal women (10, 11). In line with previous findings, we also found that E2 treatment increases the IgG-Fc sialylation as well as IgG-Fc galactosylation in an experimental set of healthy postmenopausal mice. We found that in the OVX-pla mice group, the A-galactosylated form increased compared to OVX-E2 mice, which was consistent with the recent study from Deris et al. (14). Their study investigated IgG glycome composition in pre, peri, and postmenopausal women, and found a significant drop in IgG galactosylation and sialylation particularly in postmenopausal women. During the pre- to postmenopausal transition, the A-galactosylated structure increased more than the di-galactosylated and mono-sialylated structures.

We have previously demonstrated that estrogen treatment increased the sialyltransferase, *St6gal1* expression in plasmablasts in both mice and humans, which is important for post-translational sialylation of IgG and directly influences the interaction with Fcγ receptors (15), and increased the level of sialic acid in bone marrow-derived plasma cells and moderately in B-cells (18). In this study, we used a model of postmenopausal conditions, ovariectomy, and we were unable to demonstrate any estrogen-mediated effect on sialic acid of total live cells, B-cells, or plasma cells. It is possible that for estrogens to influence immune cells, the immune system must be activated, which is not present in this study.

In contrast to IgG-Fc sialylation, postmenopausal women have increased sialylation of general serum glycoproteins (41). Despite this, we found no difference in general glycoprotein sialylation in either the long-term or short-term ovariectomized-Sham mice groups. Furthermore, we have previously reported that overall glycoprotein sialylation in the experimental ovariectomized mice did not change after immunological induction and estrogen treatment (18). Here in this study of healthy OVX mice, we found no change in sialic acid levels in general serum glycoprotein following estrogen treatment, demonstrating that sialic acid levels in serum glycoprotein are not dependent on estrogen status.

A recent study in experimental arthritis displayed that phytoestrogen treatment enhanced sialyltransferase, *St6gal1* mRNA expression in the splenic tissue (40). In contrast to the previous report, we found that E2 treatment reduced the St6gal1 mRNA expression in spleen tissue of normal OVX mice, indicating that the previously reported elevated expression of *St6gal1* is mostly due to inflammation. A downregulation in *St6gal1* mRNA at the systemic level due to the differential expression of *St6gal1* mRNA in specific cell types including immune cells and other tissue such as BM or spleen could have a distinct effect on IgG sialylation as well as potentially impact the sialylation of IgG during its circulation in the bloodstream. Sialylation of IgG may influence IgG function, half-life, and serum levels.

To summarize, we found that estrogen treatment moderately increased the IgG galactosylation, significantly increased the IgG sialylation, and decreased the A-galactosylation of IgG, in the IgG2b subclass. We were unable to confirm estrogen effects on serum IgG levels in short-term postmenopausal conditions, despite demonstrating that long-term estrogen deficiency impairs the synthesis of IgG.

## Data availability statement

The data presented in this article are deposited in the Figshare repository, accession number 10.6084/m9.figshare.23540019.

## Ethics statement

The animal study was approved by the ethics committees of the Gothenburg region, Sweden. The study was conducted in accordance with the local legislation and institutional requirements.

## Author contributions

PG and CE designed and analyzed all data. PG, TS, JaN, JoN, KH, PH, and CE performed the experiments and HC and PH provided valuable material and intellectual input. PG and CE wrote the manuscript with input from all co-authors. All authors contributed to the article and approved the submitted version

## Supplementary material

Supplementary Figure 1 Estrogen impact on bone parameters in postmenopausal mice. Mice were ovariectomized (OVX), followed by either the implantation of slow-release 17-β-estradiol (E2) or placebo (Pla) pellets (experiment-I), or subcutaneous injection of E2 or Pla, 5 days/week (experiment- II). On termination, tibia bone was collected. **(A)** Experiment-I; trabecular bone mineral density (BMD), and cortical thickness, **(B)** experiment-II; trabecular BMD, and cortical thickness. Statistical analysis was performed with One-way analysis of variance (ANOVA) followed by Dunnett’s multiple comparison tests towards OVX-Pla group in experiments-I and -II. Data are presented as scatter plots with bar graphs with ± SEM shown as vertical lines ***P* < 0.01, ****P* < 0.001.

Supplementary Figure 2 Impact of estrogen on IgG subclasses levels in normal postmenopausal mice. Mice were ovariectomized (OVX), followed by the insertion of slow-release 17-β-estradiol (E2) and placebo pellets (experiment-I), subcutaneous injection of E2 and placebo (experiment-II), and long-term estrogen-deficient (experiment-III). Serum was collected at the end of experiments and IgG1, IgG2a, and IgG2b levels in serum were analyzed: **(A)** experiment-I, **(B)** experiment-II, and **(C)** experiment-III. One-way ANOVA followed by Dunnett’s multiple comparisons to assess differences towards a placebo group in experiments-I and -II and student’s t-test were used in experiment-III. Data are presented as scattered bar graphs with mean ± SEM.

Supplementary Figure 3 Impact of estrogen on IgM levels in postmenopausal mice. Mice were ovariectomized (OVX), followed by the insertion of slow-release E2 and placebo pellets (experiment-I), subcutaneous injection of E2 and placebo (experiment-II), and long-term estrogen-deficient (experiment-III). Serum was collected on day 36 at the end of experiments- I and II, and on day 105 at the end of experiment-III. Measured IgM levels in serum: **(A)** experiment-I, **(B)** experiment-II, and **(C)** experiment-III. One-way ANOVA followed by Dunnett’s multiple comparisons to assess differences towards a placebo group in experiments-I and -II and student’s t-test were used in experiment-III. Data are presented as scattered bar graphs with mean ± SEM.
