# Supplementary material for: Impact of estrogen on IgG glycosylation and serum protein glycosylation in a murine model of healthy postmenopause
Source: Front Endocrinol (Lausanne). 2023 Sep 11;14:1243942. doi: 10.3389/fendo.2023.1243942 (PMC10519799; doi:10.3389/fendo.2023.1243942)
Supplement: Supplementary file 1 [file Table_1.pdf]

Supplementary Table 1: Detected glycoforms in murine IgG2b (peptide sequence EDYNSTIR) by LC-MS mass spectrometry analysis from experiment-II.

**Glycoform composition**[a]	**Depiction**[b]
A-galactosylated (G0)	
H3N3	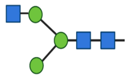
*H2N2F	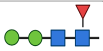
*H2N3F	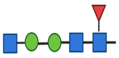
*H3N3F	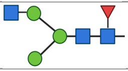
*N2F	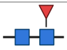
N1	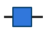
*H3N4F	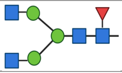
***Galactosylated* (G1/G2)**	
H4N4	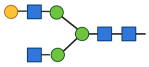
H4N3	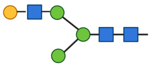
*H4N3F	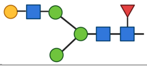
*H5N3F	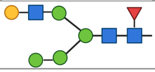
*H4N4F	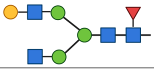
*H5N4F	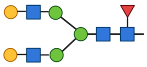
***Sialylated* (G1S/G2S)**	
*H5N3FG1	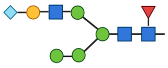
*H4N4FG1	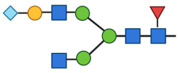
*H4N3FG1	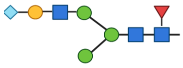
*H6N3FG1	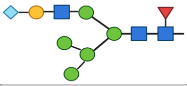
*H4N4FG1	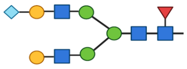
*H5N4FG2	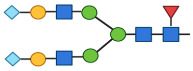

[a]H: hexose, N: N-acetyl hexosamine, F: fucose, G: N-glycolylneuraminic acid. [b]Symbols used: green circle: mannose, yellow circle: galactose, blue square: N-acetylglucosamine, red triangle: fucose, blue diamond N-glycolylneuraminic acid (Neu5Gc). *Represents the presence of fucose. The proposed glycan structures are based on fragmentation analysis and literature.
