# Supplementary figures and images for: Impact of estrogen on IgG glycosylation and serum protein glycosylation in a murine model of healthy postmenopause
Source: Front Endocrinol (Lausanne). 2023 Sep 11;14:1243942. doi: 10.3389/fendo.2023.1243942 (PMC10519799; doi:10.3389/fendo.2023.1243942)

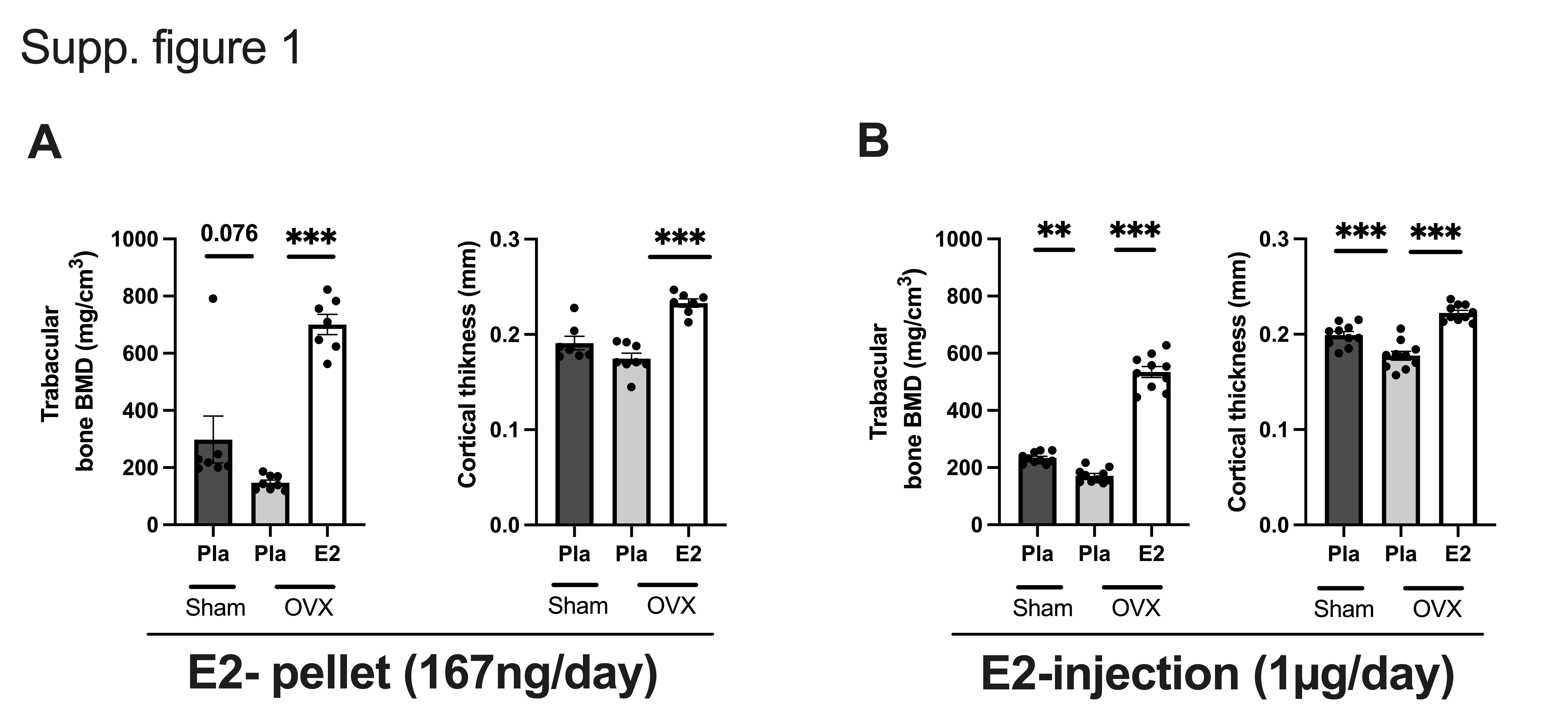

Supplement: Supplementary Figure 1 — Estrogen impact on bone parameters in postmenopausal mice. Mice were ovariectomized (OVX), followed by either the implantation of slow-release 17-β-estradiol (E2) or placebo (Pla) pellets (experiment-I), or subcutaneous injection of E2 or Pla, 5 days/week (experiment- II). On termination, tibia bone was collected. (A) Experiment-I; trabecular bone mineral density (BMD), and cortical thickness, (B) experiment-II; trabecular BMD, and cortical thickness. Statistical analysis was performed with One-way analysis of variance (ANOVA) followed by Dunnett’s multiple comparison tests towards OVX-Pla group in experiments-I and -II. Data are presented as scatter plots with bar graphs with ± SEM shown as vertical lines **P < 0.01, ***P < 0.001. [file Image_1.tiff]

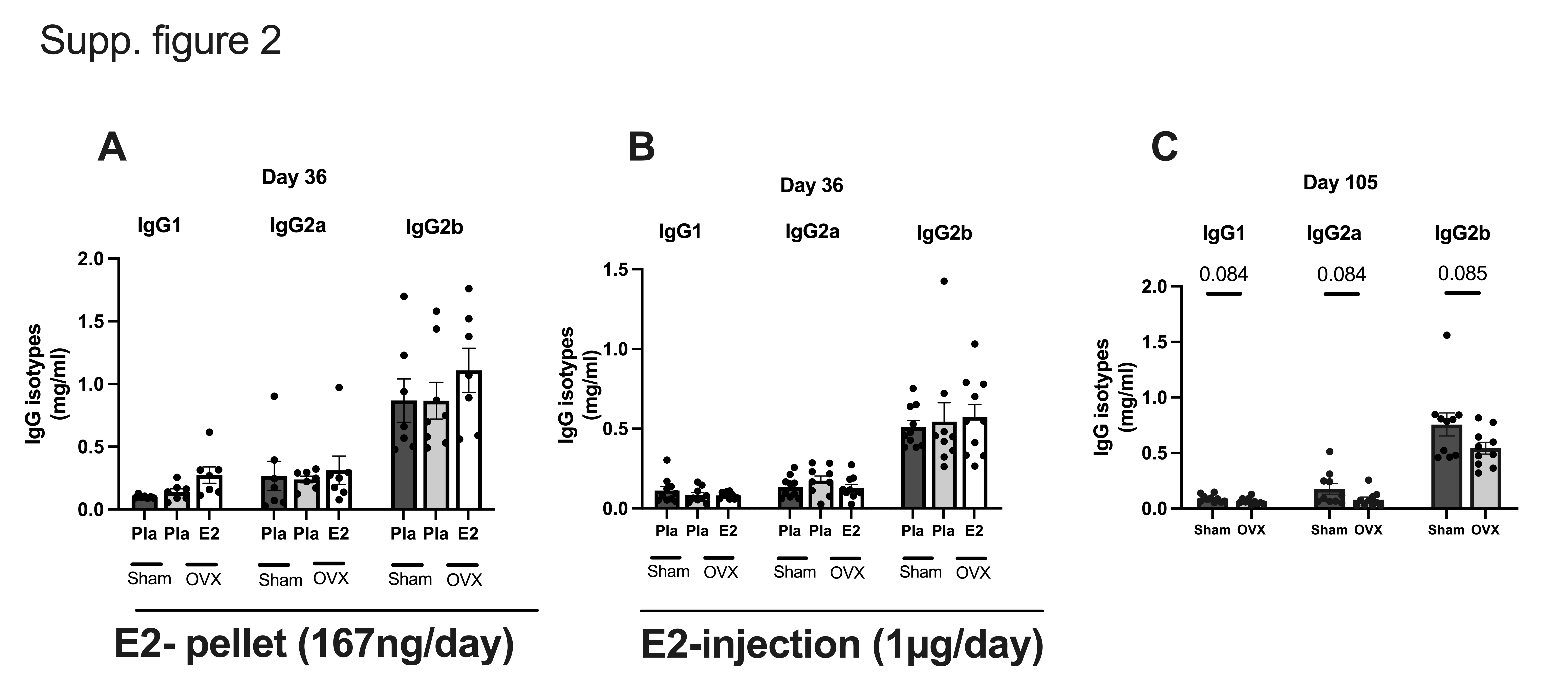

Supplement: Supplementary Figure 2 — Impact of estrogen on IgG subclasses levels in normal postmenopausal mice. Mice were ovariectomized (OVX), followed by the insertion of slow-release 17-β-estradiol (E2) and placebo pellets (experiment-I), subcutaneous injection of E2 and placebo (experiment-II), and long-term estrogen-deficient (experiment-III). Serum was collected at the end of experiments and IgG1, IgG2a, and IgG2b levels in serum were analyzed: (A) experiment-I, (B) experiment-II, and (C) experiment-III. One-way ANOVA followed by Dunnett’s multiple comparisons to assess differences towards a placebo group in experiments-I and -II and student’s t-test were used in experiment-III. Data are presented as scattered bar graphs with mean ± SEM. [file Image_2.tiff]

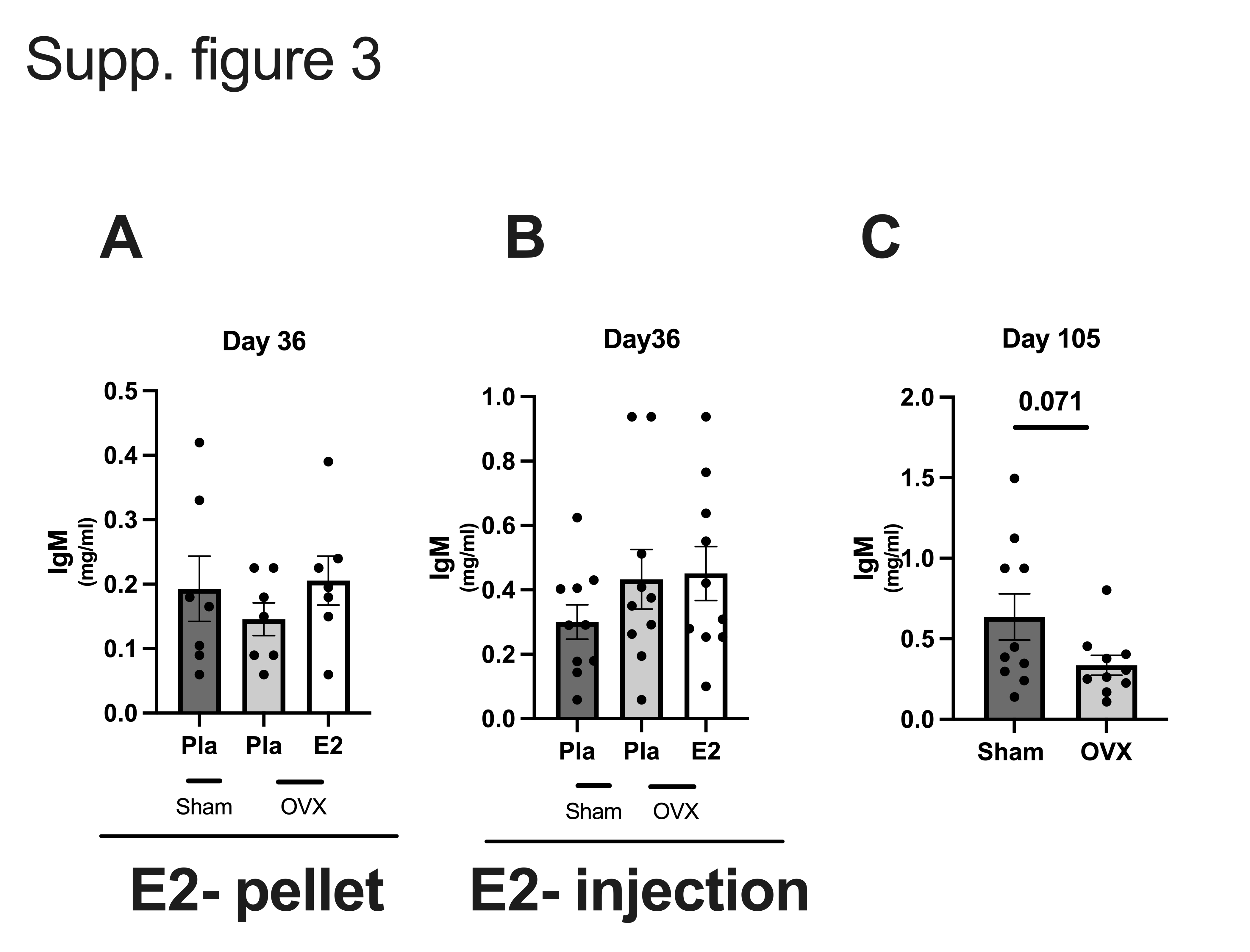

Supplement: Supplementary Figure 3 — Impact of estrogen on IgM levels in postmenopausal mice. Mice were ovariectomized (OVX), followed by the insertion of slow-release E2 and placebo pellets (experiment-I), subcutaneous injection of E2 and placebo (experiment-II), and long-term estrogen-deficient (experiment-III). Serum was collected on day 36 at the end of experiments- I and II, and on day 105 at the end of experiment-III. Measured IgM levels in serum: (A) experiment-I, (B) experiment-II, and (C) experiment-III. One-way ANOVA followed by Dunnett’s multiple comparisons to assess differences towards a placebo group in experiments-I and -II and student’s t-test were used in experiment-III. Data are presented as scattered bar graphs with mean ± SEM. [file Image_3.tiff]
